# Adipose-Derived Stem Cells as a Tool in Cell-Based Therapies

**DOI:** 10.1007/s00005-016-0394-x

**Published:** 2016-05-13

**Authors:** Anna Bajek, Natalia Gurtowska, Joanna Olkowska, Lukasz Kazmierski, Malgorzata Maj, Tomasz Drewa

**Affiliations:** 1 Department of Tissue Engineering, Nicolaus Copernicus University, Karłowicza 24, 85-092 Bydgoszcz, Poland; 2 Department of Urology, Nicolaus Copernicus Hospital, Torun, Poland

**Keywords:** Adipose-derived stem cells, Adipose tissue, Clinical trials, Regenerative medicine

## Abstract

Recent development in stem cell isolation methods and expansion under laboratory conditions create an opportunity to use those aforementioned cells in tissue engineering and regenerative medicine. Particular attention is drawn towards mesenchymal stem cells (MSCs) being multipotent progenitors exhibiting several unique characteristics, including high proliferation potential, self-renewal abilities and multilineage differentiation into cells of mesodermal and non-mesodermal origin. High abundance of MSCs found in adipose tissue makes it a very attractive source of adult stem cells for further use in regenerative medicine applications. Despite immunomodulating properties of adipose-derived stem cells (ASCs) and a secretion of a wide variety of paracrine factors that facilitate tissue regeneration, effectiveness of stem cell therapy was not supported by the results of clinical trials. Lack of a single, universal stem cell marker, patient-to-patient variability, heterogeneity of ASC population combined with multiple widely different protocols of cell isolation and expansion hinder the ability to precisely identify and analyze biological properties of stem cells. The above issues contribute to conflicting data reported in literature. We will review the comprehensive information concerning characteristic features of ASCs. We will also review the regenerative potential and clinical application based on various clinical trials.

## Introduction

In the past, adipose tissue was considered only a passive energy storage. Since the mid-80’s of the last century, when its participation in the metabolism of sex hormones was confirmed, adipose tissue has become an important endocrine organ that controls metabolism, immunity and satiety (Seo et al. 2004). The real breakthrough was made in 2001 when a new source of adult stem cells, called adipose-derived stem cells (ASCs), isolated from adipose tissue, was described for the first time (Zuk et al. 2001).

Adipose tissue is considered a type of connective tissue exhibiting morphological, functional and regulatory heterogeneity (Furstenberg et al. 2010). The interaction between adipose tissue and other tissues or organs is certainly bidirectional (Skowrońska et al. 2005). It affects the functioning of most physiological systems, including the immune, reproductive, and hematopoietic system (Baptista et al. 2015). Development, amount and distribution of adipose tissue in the body is a result of the nervous system, hormones and transcription factors effects (Baptista et al. 2015; Furstenberg et al. 2010). In terms of macroscopic features five types of adipose tissue [bone marrow (BM), brown and white mammary glands and mechanical adipose tissue] can be distinguished, each of them with a different, distinct biological function (Gimble et al. 2007).

Since the identification of stem cells in the BM over 40 years ago, they have become a standard in the field of tissue engineering and regenerative medicine (Friedenstein et al. 1968; Lindroos et al. 2011). Bone marrow aspiration, however, is a painful and expensive procedure that requires general anesthesia. Moreover, the yield of isolated cells is usually low (Lindroos et al. 2011). Multipotent mesenchymal stem cells (MSCs) are present in a number of postnatal organs and connective tissues. MSCs, with similar characteristics to BM-derived MSCs, have been successfully isolated from different tissue sources including periosteum, synovium, skeletal muscle, skin, peripheral blood and umbilical cord (Kuroda et al. 2011). Although the stem cell populations derived from these sources are valuable, common problems include low harvested cells yield and limited amount of harvested tissues. Adipose tissue seems to be a great alternative source of acquiring stem cells, especially with the increased incidence of obesity which makes the tissue abundant and readily accessible. It can be collected in large quantities with low possibility of donor site morbidity, during a much safer and less time consuming liposuction procedure (Uzbas et al. 2015). Approximately 400,000 liposuction surgeries are performed in the US each year and these procedures yield from 100 ml to >3 l of lipoaspirate tissue, which is routinely discarded (Bunnell et al. 2008). Furthermore, the high content of ASCs in adipose tissue, excludes the need of long-term in vitro culture, what reduces the risk of chromosomal abnormalities. Such properties make the ASCs an attractive tool in clinical application for therapeutic purposes (Baptista et al. 2015; Nakagami et al. 2006; Uzbas et al. 2015).

## Collection of Adipose Tissue: Liposuction

The method of adipose tissue collection, for the purpose of stem cell isolation, raises a number of controversies. Researchers are eager to use adipose tissue, which is considered a waste product of abdominoplasty, cosmetic surgery or liposuction (Gimble et al. 2010). Liposuction is currently the most common aesthetic surgery widely used around the world. The first step is the infiltration of the fat (of the area to be aspirated) using a large amount of the lidocaine, epinephrine, sodium bicarbonate, and saline composition (to reduce the risk of blood loss). Infiltration methods include the super-wet technique (1 ml infiltration per 1 ml aspiration) and the tumescent technique (2–3 infiltration cm^3^ per 1 cm^3^ aspiration) (Heymans et al. 2006). Subsequently the separated fat tissue with the injected solution is aspirated via a cannula of different diameter (Sood et al. 2011; Vivek et al. 2014).

Several liposuction techniques such as standard liposuction, suction assisted liposuction, internal ultrasound assisted liposuction, external ultrasound assisted liposuction, laser assisted liposuction, power-assisted liposuction, and vibroliposuction are currently in clinical use (Heymans et al. 2006). A brief description of selected methods is shown in Table 1.

**Table 1:** Characteristic of selected liposuction methods

Technique	Characteristic
Standard and suction assisted liposuction	Aspirating the tissue at high pressure with a large diameter cannula without prior infiltration (dry technique, now rarely used)
Tumescent techniques	
Ultrasound-assisted liposuction	Emulsifying the fat tissue with a vibrating at high ultrasound probe, emulsion removal by the small diameter cannula
Power-assisted liposuction	The separation of adipose tissue by the set forward and backward motion cannula, divided fat tissue is sucked into the cannula, considered as the safest and most effective method
Laser-assisted liposuction	Adipocytes separation and liquidation of tissue via a cannula emitting the laser ray, gentle aspiration of the liquid fat tissue through the cannula

Modified according to Heymans et al. (2006)

There are several liposuction techniques currently available to remove fatty tissue from different sites on the human body. In the tumescent techniques, that reduce post operative bruising, swelling and pain in comparison to older more traditional methods, subcutaneous fat is injected with a large volume of diluted anesthetic and capillary constrictor before liposuction is performed

Depending on the technique and location, the liposuction procedures are performed under local, peripheral or general anesthesia (Sood et al. 2011). Liposuction is regarded as a generally safe and well-tolerated procedure with minimal post-operative discomfort. The study performed by the American Society for Dermatologic showed no recorded deaths at nearly 70,000 liposuction procedures carried out between 1994 and 2000, and serious adverse events were very rare at the rate of 0.68 per 1000 cases (Fraser et al. 2006). Compared to the BM aspiration, liposuction procedures are much less invasive, cheaper, and even a small amount of adipose tissue (100–200 ml) is enough to isolate a suitable number of stem cells under local anesthesia (Orbay et al. 2012). Other methods such as syringe-based harvest may give higher yields of adipocytes but are not as widely used as vacuum liposuction (Eto et al. 2009). Surgical fat excision being more invasive may be used less often but the ASC’s yield is over 50 % higher compared to liposuction (Fraster et al. 2007).

## The Stromal Vascular Fraction

Isolation procedures of fat tissue components are convergent in the standard methods, based on enzymatic digestion. Those methods are designed to separate the two easily recognizable fractions, mature adipocytes and the stromal vascular fraction (SVF). After collagenase digestion, mature adipocytes with a high fat content are separated as a floating layer. All cells, which remain after the removal of mature adipocytes constitute the SVF (Peinado et al. 2012). The composition of the SVF varies among individual research groups. It is commonly known, however, that it includes preadipocytes, endothelial cells, pericytes, fibroblasts, ASCs and hematopoietic stem cells (Peinado et al. 2012; Zuk et al. 2001). The SVF also contains erythrocytes, lymphocytes T, B cells, macrophages, and mast cells (Peinado et al. 2012). Stem cells and progenitor cells determine about 3 % of all cell populations (Tsuji et al. 2014).

Cytometric analysis performed by Yoshimura et al. (2006) identified the following cell populations in SVF: ASCs (CD31^−^/CD34^+^/CD45^−^/CD90^+^/CD105^−^/CD146^−^), endothelial progenitor cells (CD31^+^/CD34^+^/CD45^−^/CD90^+^/CD105^low^/CD146^++^), pericytes (CD31^−^/CD34^−^/CD45^−^/CD90^+^/CD105^−^/CD146^+^) and blood cells (CD45^+^), suggesting also the presence of fibroblasts, vascular smooth muscle cells and preadipocytes. Taking into account the surface markers expression, the heterogeneous isolates of SVF can be represented as follows: 11 % CD2^+^ cells, 18 % CD11a^+^ cells, 29 % CD14^+^ cells, 49 % CD31^+^ cells, 57 % CD45^+^ cells and 60 % CD90^+^ cells (Brzoska et al. 2005). Another research group presented composition of SVF slightly different: 11 % CD14^+^ cells, ~2 % CD31^+^ cells, ~7 % CD34^+^ cells, ~9 % CD45^+^ cells, ~29 % CD90^+^ cells and ~47 % CD146^+^ cells (Astori et al. 2007). It was also observed that 85 % SVF cells, which underwent adhesion, exhibit the CD31^−^/CD34^+^/CD45^−^/CD105^+^ phenotype (Varma et al. 2007). The expression of hematopoietic markers such as CD11, CD14, CD34, and CD45 dissipate is lost during the SVF in vitro passaging. On the other hand, the expression level of some markers, e.g. CD29, CD73, CD90, and CD166 increases in higher passages (Tsuji et al. 2014). That is why passaging is considered a selecting factor when it comes to cell populations with more homogenous cell surface markers compared to SVF.

## Isolation of ASCs from Adipose Tissue

For the first time the isolation of rat mature adipocytes and adipose tissue progenitor cells was described by Rodbell (1964). The protocol was based on the disintegration of adipose tissue into small fragments followed by enzymatic digestion with collagenase type I in 37 °C and the following centrifugation to separate distinct cell fractions. The obtained supernatant was composed of mature adipocytes and the pellet fraction consisted of the SVF components, in which the progenitor cells of adipocytes were presumably present. This procedure was effectively modified and used with small volumes of fat tissue by many research groups like (Cawthorn et al. 2012; Tsuji et al. 2014).

Katz et al. (1999), Zuk et al. (2001) and Uzbas et al. (2015) demonstrated that SVF isolated from human lipoaspirates contains multipotent cells. To isolate these cells enzymatic digestion was used followed by their adhesion to the bottom of culture flasks. Enzymatic digestion and adhesion properties of ASCs still comprise the basis of their isolation (Lindroos et al. 2011). One of the novel methods of ASCs isolation is the use of antibody-coated immunomagnetic beads, which are able not only to “catch” ASCs cells, but also other subpopulations of stem cells found in adipose tissue (Rada et al. 2009).

Katz et al. (2001) developed a device known as the “bag within a bag”, which enables the aspiration of pre-injected saline and tissue to the inner bag. This system is extremely useful for contamination removal and helps to automate the isolation protocol to some degree (Katz et al. 2001). The same group developed a rotary, temperature control device to digest even 1 l of tissue at a time (Katz et al. 1999). In addition to the cell isolation it also separates the extracellular matrix of adipose tissue, which may be used as a potent biomaterial.

Isolation of ASCs on a large scale is possible with the use of commercially available systems, such as CelutionTM (Cytori Therapeutics, San Diego, USA) (Lin et al. 2008b; Tissue Genesis Incorporated 2009). These systems allow for fast and safe isolation of large numbers of autologous stem cells, ready for immediate application. CelutionTM Cytori system was used in a clinical trial of stress urinary incontinence treatment, which showed promising results (Yamamoto et al. 2010).

The most important advantage of ASCs is their abundance. From 1 g of adipose tissue an average of 0.5–2.0 × 10^6^ SVF cells can be isolated, which gives 1–10 % of stem cells yield (Bear et al. 2013) for comparison MSCs constitute 0.001–0.01 % of BM (Pittenger et al. 1999). Some sources claim that the number of isolated cells can be even greater, and amount to about 2–6 × 10^6^ cells from 1 ml of tissue (Kim and Jeong 2014). The number of ASCs isolated from 1 g of fat tissue ranges between 0.5 × 10^4^ and 2 × 10^5^ (Baer and Geiger 2012). These differences are caused by donor characteristics, such as gender, age, ethnicity, BMI, disease history and also the type of fat tissue (yellow/brown), location (subcutaneous/visceral fat) and the tissue collection method or culture conditions (Baer and Geiger 2012; Olkowska-Truchanowicz 2008).

## Impact of Culture on Senescence and Genetic Stability of ASCs

The protocol of ASCs culture varies between laboratories and currently there is not a single, unified method available. Most commonly it is a monolayer culture in standard medium supplemented with 10 % fetal bovine serum (Mizuno et al. 2012). The growth factors can affect the undifferentiated state of ASCs. Under in vitro conditions stem cells phenotype may be affected by growth medium used for ASC culture, specific supplements added to basic growth medium and environment conditions at which cells are grown. Even the use of serum-containing standard medium may induce their differentiation. Under in vitro conditions ASC’s phenotype may be affected by a wide variety of factors, ranging from growth medium choice and the type of supplementation used (serum, platelet lysate, growth factors, corticoids, antioxidants) to environment and culture conditions such as culture perfusion, mechanical load, confluence level or substance stiffness. ASC’s are also especially sensitive to oxygen levels, if possible complete hypoxia condition would be best for their culturing (Baer and Geiger 2012).

In comparison to BM-MSCs, ASCs have a higher proliferative capability. The population doubling time, in the logarithmic growth phase, ranges 40–120 h and depends on age of the donor, type and location of the adipose tissue, method of collection, culture conditions, cells density and medium composition used by teams of De Ugarte et al. (2003), Izadpanah et al. (2006), Mitchell et al. (2006) and Zuk et al. (2001). ASCs show telomerase activity, and although it is lower than in the tumor cell lines, it testifies the ability of ASCs for self-renewal and proliferation (Jeon et al. 2011). These cells maintain telomere length during long-term culture, but whether telomerase activity is sustained at a constant level or decreases with successive passages is still open to debate (Izadpanah et al. 2006; Jeon et al. 2011). Sachs et al. (2012) documented the lack of telomerase expression in ASCs. Moreover, telomeres were gradually shortening with age, indicating a telomere-based senescence mechanism (Sachs et al. 2012).

Taking into account that stem cells previously cultured in vitro can be safely transplanted, there is a great need to further investigate their abilities to maintain chromosome stability in non-physiological conditions. ASCs exhibit higher genetic stability in long-term culture compared to BM-MSCs (Dahl et al. 2008; Neri et al. 2013). However, research carried out by at least one research group has shown that karyotype irregularities occur with a frequency of >30 %. The occurrence of neoplastic transformation of ASCs maintained in culture for more than four months was also observed (Rubio et al. 2005). The relevance of a clear tendency towards increased aneuploidy with in vitro culture is still being debated (Sensebe et al. 2012). It appears crucial to perform a detailed analysis of the genome prior to any cell-based treatment. Special caution in conducting and selecting the ASCs culture is needed, as it can be crucial for maintaining their diplioidal karyotype.

## Phenotype and Niche of ASCs

All previous attempts to determine the phenotype of ASCs and identification of one unique marker for these cells were unsuccessful. It is assumed that these cells express following markers: CD13, CD29, CD44, CD49b, CD90, CD105 and do not express the hematopoietic markers, such as CD14, CD31, CD45 and CD144 as described by Zuk (2013). Additional studies, evaluating the ASCs differentiation, suggested that these cells have a specific expression of CD34 marker and the absence of CD31/CD45 (Varma et al. 2007; Yoshimura et al. 2006). The expression profile of cell surface markers of human ASCs is presented in Table 2.

**Table 2:** The expression profile of ASCs surface markers

Protein	Name	CD
Positive expression		
Adhesion molecules	Tetraspan protein	CD9
	β-1 integrin	CD29
	Sialomucin	CD34
	α-4 integrin	CD49d
	Intercellular adhesion molecule-1	CD54 (ICAM-1)
Receptor molecules	Hyaluronan receptor	CD44
	Transferrin receptor	CD71
	α-Platelet-derived growth factor	CD140a
Enzymes	Neutral endopeptidase	CD10 (CALLA)
	Aminopeptidase	CD13
	Ecto-5′-nucleotidase	CD73
Extracellular matrix proteins and glycoproteins	Collagen I	–
	Collagen III	–
	Osteopontin	–
	Osteonectin	CD90
	Thy-1	
Cytoskeletal proteins	Vimentin	–
Regulatory proteins of the complement system	Complement decay-accelerating factor	CD55
	MAC-inhibitory protein	CD59
Histocompatibility proteins	HLA-A, -B, -C (class I)	–
Negative expression		
Adhesion molecules	Vascular cell adhesion protein 1	CD106 (VCAM)
	Lymphocyte function-associated antigen 1	CD11a
	Mac-1a	CD11b
	Integrin α-X	CD11c
	Platelet endothelial cell adhesion molecule	CD31
	VE-cadherin	CD144
Receptor molecules	LPS receptor	CD14
Enzymes	Tyrosine phosphatase	CD45
Controversy expression		
Adhesion molecules	Endoglin	CD105
	MUC-18	CD146
	Activated leukocyte cell adhesion molecule	CD166 (ALCAM)
Receptor molecules	Platelet derived growth factor receptor	CD140b
Muscle proteins	Smooth muscle actin	–
Histocompatibility proteins	HLA-DR	–

Modified according to Zuk (2013)

Adipose-derives stem cells exhibit specific cell surface expression pattern. Despite intensive search single unique marker have not been identified. ASCs are mainly identified on the basis of their high expression of CD105, CD90, CD44, CD73 in the absence of CD34, CD14 and CD45

Stem cell niche is a microenvironment, which controls the properties of stem cells and gene expression. It consists of signaling molecules, interactions between cells themselves, stem cells and the extracellular matrix (Fuchs et al. 2004). The niche of ASCs in adipose tissue has not been clarified. However, a number of recent studies indicate that it can be identified in adipose tissue vascularization process like shown by (Leto Barone et al. 2013; Lin et al. 2008a; Zimmerlin et al. 2010). Zimmerlin et al. (2010) identified the ASCs on outer adventitia of blood vessels. Expression of CD146 and 3G5 and the pericytes markers suggests that these cells can be identified as pericytes located in the vasculature of adipose tissue (Crisan et al. 2008; Zannettino et al. 2008). This hypothesis may enhance the fact that processes like adipogenesis and angiogenesis are closely related, adipose tissue is a highly vascularized tissue, and pericytes exhibit similar multipotency as stem cells (Crandall et al. 1997; Feng et al. 2010; Zannettino et al. 2008).

Expression of CD146 by ASCs, however, raises a lot of doubt. It also impedes the possibility of confirmation that pericytes and ASCs are the same cells (Sensebe et al. 2012). Moreover, Maumus et al. (2011) in immunohistological analysis presented that native ASCs exhibit specific morphological characteristics. In addition, native ASCs can be found in the adipose tissue matrix. An argument against the classification of ASCs as pericytes militates is the fact that they do not express markers characteristic to pericytes in vivo, such as NG2, CD140b or alpha smooth muscle actin (expression occurs during in vitro culture) (Casteilla et al. 2011). Rodeheffer et al. (2008) identified another subpopulation of early adipocyte progenitor cells in a mice model (Lin^−^, CD29^+^, CD34^+^, Sca-1^+^, CD24^+^), which is located in white adipose tissue.

The cause of contradictions concerning the profile of ASCs is not known. It may have emerged as a result of various isolation and cultivation methods used or various location-dependent origins of ASCs (Casteilla et al. 2011). The presence of several stem cell populations within SVF of adipose tissue, such as endothelial precursor cell population, population of perivascular ASCs or pericytes population can also be the reason of that problem (Tsuji et al. 2014; Zeve et al. 2009).

## Mechanisms Responsible for Regenerative Properties of ASCs

Several mechanisms can be responsible for the regenerative potential of ASCs. Some studies suggest that ASCs act via differentiation towards a specific cell type, thereby replacing defective cell populations in vivo. However, studies conducted on animal models, which allow for tracking ASCs in vivo do not confirm the aforementioned hypothesis (Gimble et al. 2012). With an exception of the “classic” mesenchymal phenotype (differentiation into adipocytes, osteoblasts, chondrocytes) none of the studies confirm the total and functional differentiation of mesenchymal-like cells. The phenotype of differentiated cells is mostly confirmed only by the expression of specific markers (Casteilla et al. 2011).

Some studies suggest that MSCs differentiation may lead to “intermediate two-phenotypic cells”, which show co-expression of specific cells and stromal markers without obtaining actual functionality (Rose et al. 2008). In such case, MSCs undergoing a specific differentiation would only express markers of “truly differentiated cells”, without demonstrating all of their functionality. Another unresolved problem is whether cells transdifferentiation is a result of their fusion which is considered to be the main mechanism for the formation of new functional cells (Utsunomiya et al. 2011). In a study conducted by Aurich et al. (2009), there was no detected fusion of the implanted ASCs, differentiated towards hepatocytes, with hepatocytes of the host.

Another proposed mechanism of ASCs activity is the modulation stem cell niche of the host by stimulating the recruitment of endogenous stem cells to the damaged area and their commitment in the proper lineage. ASCs may be also a source of antioxidants, free radical scavengers and chaperone/heat shock proteins at the site of tissue damage. As a result of their actions, toxic substances are separated, removed and the surviving cells can restore their function (Friedenstein et al. 1968). Currently, many researchers believe that ASCs promote cell regeneration of tissues and organs, primarily through the release of cytokines and growth factors (Gimble et al. 2012).

## Paracrine and Immunomodulatory Properties of ASCs

ASCs promote tissue regeneration by secreting cytokines and growth factors that stimulate restoration of normal tissue function or reduce its damage. Molecules secreted by ASCs have a positive effect on the central nervous system, immune system, heart, muscles and even the general vitality of cells (Salgado et al. 2010).

ASCs cell cytokine profile (Table 3) comprises, inter alia, vascular endothelial growth factor (VEGF), granulocyte/macrophage colony-stimulating factor, cell-derived stromal factor 1-alpha, hepatocyte growth factor (HGF), transforming growth factor β, and fibroblast growth factor 2, which explains their impressive angiogenic properties and ability to induce tissue neovascularization (Gir et al. 2012; Sterodimas et al. 2010). Among the secreted anti-apoptotic factors it is worth to mention about insulin-like growth factor-1 (IGF-1), which, as was shown, may protect the cardiomyocytes against apoptosis (Sadat et al. 2007).

**Table 3:** ASCs cytokine profile

Function	Secreted protein
Immunomodulation	TGF-β, HGF, PGE2, IL-6
Vascularization	VEGF, HGF, TGF-β2, FGF-2, bFGF, GM-CSF
CUN regeneration	BDNF, NGF, GDNF, IGF-1
Hematopoiesis suport	HGF, GM-CSF, IL-6,7,8,11, TNF-α
Other	Adiponectin, angiotensin, cathepsin D, retinol binding protein, CXCL12

Modified according to Salgado et al. (2010)

Adipose-derived stem cells secrete several cytokines and growth factors that modulate immune response and facilitate regeneration of damaged tissues. These soluble mediators exhibit positive effect on central nervous system, heart, muscles and even the general vitality of cells

*GM-CSF* granulocyte/macrophage colony-stimulating factor, *TGF-β* transforming growth factor β, *FGF-2* fibroblast growth factor 2, *BDNF* brain derived neurotrophic factor, *GDNF* glial derived neurotrophic factor, *NGF* nerve growth factor

ASCs promote the regeneration of central nervous system cells and show a neuroprotective activity by secretion of brain derived neurotrophic factor, glial derived neurotrophic factor, nerve growth factor and IGF (Salgado et al. 2010). There is also evidence that growth factors, secreted by ASCs, stimulate the growth of fibroblasts and keratinocytes (Hong et al. 2013). In response to inflammatory stimuli, derived from adipose tissue, expression of angiogenic factors (VEGF, HGF, IGF-1), and hematopoietic/inflammatory factors (G-CSF, M-CSF, IL-6, TNF-α) in ASCs is increased (Kilroy et al. 2007).

ASCs are also immunoprivileged due to the lack of HLA-DR expression and the proliferation inhibition of activated allogeneic lymphocytes (Aust et al. 2004; Gonzalez-Rey et al. 2010; Mitchell et al. 2006). ASCs inhibit the generation of pro-inflammatory cytokines, stimulate the production of anti-inflammatory IL-10 cytokine and induce the formation of antigen-specific regulatory T cells (Gonzalez-Rey et al. 2010). The immunosuppressive properties of ASCs also result from the production of prostaglandin E2 and 2,3 dioxygenase indole (Gimble et al. 2011). These cells also protect against organ rejection and prevent from graft versus host disease after allogeneic stem cell transplantation (Yañez et al. 2006). Immunomodulatory properties have been confirmed both in vitro and in vivo (Baer 2014; Le Blanc et al. 2003; Nagaya et al. 2014; Patel et al. 2008).

## Multilineage Differentiation Potential of ASCs

Literature provides abundant evidence concerning the in vitro multipotency of ASCs. Furthermore, this property is maintained during long-term culture (Baer and Geiger 2012). It is generally believed that ASCs origin from mesoderm, therefore, their potential to differentiate towards adipocytes, chondrocytes, osteoblasts and myocytes should be obvious and was confirmed in many studies (Mizuno 2009). Induction of ASCs differentiation in vitro occurs mainly by culturing cells in culture media supplemented with specific growth factors (Baer and Geiger 2012). Subsequent studies have expanded the potential of adipose derived stem cells on the ability to differentiate into non-mesodermal cells, i.e. ecto- and endodermal (Mizuno 2009).

ASCs support hematopoiesis and angiogenesis, also their differentiation potential toward endothelial cells and their participation in the blood vessels formation is confirmed in literature (Sood et al. 2011). Aforementioned cells cultured in vitro on the matrigel quickly and easily form a vascular-like structure adopting the endothelium function (Cao et al. 2005; Sood et al. 2011). Formation of the functional vascularization by these cells was confirmed in vivo in a number of models such as: myocardial infarction, regeneration of epithelium and nerve tissue (Baptista et al. 2015).

Some reports about the possibility of ASCs differentiation into the insulin-producing β cells, glucagon and somatostatin appeared in literature (Colazzo et al. 2010). ASCs were able to differentiate towards hepatocyte-like cells, expressing albumin and α-fetoprotein, LDL uptake and urea production (Lindroos et al. 2011). In vivo, hepatocyte-like cells derived from ASCs reconstitute the function of hepatocytes (Timper et al. 2006).

Findings concerning the ASCs participation in the formation of functional neurons are contradictory. Some studies confirm their differentiation into neuronal cells, both morphologically and functionally (Seo et al. 2005). Many researchers see hope in treatment of nerve injuries using ASCs thus, confirming their participation in neuronal regeneration (Mizuno et al. 2012; Khalifian et al. 2015; Zack-Williams et al. 2015).

However, in most cases, the analysis of ASCs multipotency is based, on morphology and surface marker expression of differentiated cells in vitro (Di Summa et al. 2010). Only a hand full of studies evaluate the differentiation effect in terms of functionality, such as the myocytes contractility (Johal et al. 2015; Rangappa et al. 2003). Currently the main focus of researchers regarding the potential of in vivo transplanted ASCs lies in the context of tissue engineering and regenerative medicine (Table 4) (Di Summa et al. 2010).

**Table 4:** In vitro and in vivo multipotency of ASCs

	Differentiation inducing factors	Differentiation in vitro	Differentiation in vivo	Examples
White adipocytes/adipose tissue	Insulin, IBMX, dexamethasone, indometacin, rosiglitazone, thiazolidinedione	Yes	Yes	Gelatin matrix + preinduced human ASCs, subcutaneous, mice lacking thymus Fibrin glue + GFP + murine visceral ASCs, subcutaneous, mouse
Osteoblasts/bone	25-Dihydroxyvitamin D3, glycerophosphate, ascorbic acid, bone morphogenetic protein-2, valproic acid	Yes	Yes	PLGA scaffold + human ASCs induced toward osteoblasts, skull damage, rats lackingthymus PLA scaffold + rat ASCs induced toward osteoblasts, palate damage, rats
Chondrocytes/cartilage	FGF, TGF-β, dexamethasone, IGH, bone morphogenetic protein-6, ascorbic acid, insulin	Yes	Yes	PLGA scaffold + induced with TGF-β1 human ASCs, subcutaneous, mice lacking thymus Fibrin glue + rabbit ASCs, cartilage damage, rabbits
Skeletal myocytes	Dexamethasone, horse serum, co-culture with primary myoblasts or myoblast cell lines	Yes	Yes	Preinduced human ASCs + mouse model of muscular dystrophy
Cardiomyocytes	Interleukin-3, interleukin-6, transferrin, VEGF, 5-azacytidine	Yes/No	Yes	ASCs + injection into the myocardial, infarction model, rats lacking thymus Cardiomyoblasts derived from human ASCs + porcine model of myocardial infarction
Smooth muscle myocytes	Heparin, TGF-β1, bone morphogenetic protein-4	Yes	Yes	ASCs co-culture with human bladder smooth muscle + injection into the bladder, mice lacking thymus Rats ASCs, model of stress urinary incontinence, rats
Endothelial cells	EGF, bFGF, hydrocortisone	Yes/no	Yes	Human ASCs + model of hindlimb ischemia, mice Rats ASCs + model of ischemia–reperfusion injury, rats, intravenous administration
Nerve cells	Valproic acid, insulin, hydrocortisone, EGF, FGF	Yes/no	Yes	Schwann cells derived from ASCs + rat spinal cord injury, rats ASCs induced toward neurogenesis + rat focal brain injury, rats
Hepatocytes	HGF, oncostatin, dimethylsulfoxide	Yes	Yes	Human ASCs + partial hepatectomy, mice lacking thymus, intravenous Human ASCs + CCl4-induced liver injury, mice lacking thymic
β cells pancreatic islet	Nicotinamide, activin-A, exendin-4, HGF, pentagastrin, glucose	Yes	Yes	Rats ASCs + Pdx1 transduction, streptozotocin-induced diabetes, rats

Modified according to Cawthorn et al. (2012), Hoekstra (2011), Yarak and Okamoto (2010) and Zuk (2013)

Adipose derived stem cells have potential to differentiate into cells of mesodermal and non-mesodermal origin. When maintained under optimal growth conditions they have been shown to be multipotent, capable of differentiating down the adipogenic, osteogenic and chondrogenic lineages. Differentiation into other cell types, i.e. cardiomyocytes or endothelial cells was also confirmed

## Clinical Studies with ASCs

In clinical applications, ASCs were used for the first time in year 2004, when they were implanted together with a bone fragment, fibrin glue and a biodegradable scaffold to regenerate a large bone defect in the skull of 7-year-old girl (Hoekstra 2011). Currently, the quantity of proposals for the use of these cells in tissue repair and regeneration is impressive. Number of clinical trials evaluating the efficacy and safety of ASCs in tissue reconstruction and regeneration increases significantly each year.

According to the clinical trials database (ClinicalTrials.gov database 2015) there are 122 studies currently registered as using adipose derived stem cells. These studies include treatment of: diabetes, liver cirrhosis, fistulas, cardiovascular disease, limb ischemia, amyotrophic lateral sclerosis, lipodystrophy, graft versus host disease, Crohn’s disease, atherosclerosis, soft-tissue augmentation and bone defects (Table 5) (Lendeckel et al. 2004). As Kim and Jeong (2014) stated in his review, ACSs are more liberally used in clinical treatment in Korea and Japan for plastic surgery and esthetic treatments because of legal reasons. Most of currently conducted research is at its early stages (Table 6), and the study population do not not exceed 100 patients. A large amount of studies contains only a descriptions of cases rather than randomized trials, without any control groups. Five studies were terminated (Table 7), including the cirrhosis treatment due to a lack of efficacy.

**Table 5:** Clinical trials with the use of ASCs

Indication	Number of trials
Endocrine system	
Diabetes and its complications	6
Gastrointestinal and urogenital tract	
Crohn’s disease/fistula/faecal incontinence	19
Urinary incontinence	3
Renal failure	1
Liver failure	3
Ovaries failure	1
Eractile dysfunction/urethral structure	4
Ischemia	
Angiogenesis/Burger disease/limb ischemia	12
Vascular occlusive disease of the kidney	1
Myocardial infarction	4
Hard and soft tissues	
Arthritis/bone/cartilage	24
Lipodystrophy	2
Romberg disease	1
Cosmetics reconstruction	7
Other	
Central nervous system/keratopathy/multiple sclerosis/Parkinson’s disease/stroke	11
GvHD	1
Spinal cord injury	4
Autism	1
Heart failure	4
Frailty syndrome	1
Chronic obstructive pulmonary disease/pulmonary fibrosis	5
Dry macular degeneration/retinal degeneration	2
Pain	2
Acute respiratory distress syndrome	1
Sepsis	1

On the basis of (ClinicalTrials.gov database 2015)

Adipose derived stem cells are currently used in several clinical trials. Most of them concern gastrointestinal and urogenital tract pathologies and cartilage and bone degeneration

*GvHD* graft versus host disease

**Table 6:** Phase of clinical trials with the use of ASCs

Study chase	Number of the studiem
0	2
I	23
II	18
I/II	58
III	5
II/III	1
IV	2
Unknown	13

On the basis of (ClinicalTrials.gov database 2015)

Most of ongoing clinical trials evaluating regenerative potential of adipose-derives stem cells are at early stage of the study. Their main goal is to assess stem cell therapy safety within small groups of patients and identify its side effects

**Table 7:** Status of clinical trials with the use of ASCs

Study status	Number of trias
Not recruiting	32
Recruiting	60
Finished	25
Interrupted	5

On the basis of (ClinicalTrials.gov database 2015)

Majority of clinical trials evaluating efficacy of stem cell therapy are at early stage of patient recruitment. Only 20 % were finished. Some studies were interrupted including cirrhosis treatment due to lack of efficacy

Clinical studies are generally based on the use of the whole SVF, isolated ASCs (alone or in combination with biomaterials) or fat tissue itself enriched with ASCs. Studies differ in the ASCs type used, some use autologous ASCs, other allogenic ones. There is also a significant difference in density of transplanted cells and the route of their administration. It is also worth to note that some studies are based on the immunomodulatory and angiogenic properties of ASCs. These studies concern the treatment of autoimmune diseases, ischaemia or diabetic wounds of lower limbs. Researches based on ASCs differentiation, include treatment of degenerative arthritis, cardiac or spinal injury.

## Why the Cell-Based Therapies Can Fail?

The development of tissue engineering, regenerative medicine and cell based therapies will surely prevent many currently incurable diseases. However, before achieving an efficient clinical application it is necessary to overcome many technical limitations. There are certainly a lot of questions that should be addressed at preclinical and clinical levels, e.g. spontaneous differentiation of stem cells into target cells or the problem of cell migration and homing mechanisms. We have to consider adipose tissue as an effective regeneration tool, isolation of a homogenous stem cell fraction is crucial. Recently, a whole adipose tissue-derived SVF became popular in clinical applications, especially in one-step surgeries in the field of orthopedics. However, there is little data concerning a full characterization of this fraction, which is very heterogeneous. It contains the mixture of endothelial cells, smooth muscle cells, fibroblast, pericytes and mast cells. In such therapies we transplant the whole microenvironment of stem cells rather than just stem cells alone. Therefore it is not surprising that grafts with the SVF fraction give promising results. However, we need to understand the mechanism of action before introducing it to common clinical practice. There is a huge need to answer which factors give and support the best therapeutic effect, especially in long-term basis. Further questions arise, whether the grafted stem cells can maintain their undifferentiated state or do they differentiate. This last issue is very important in the light of a possibility of undesirable ASCs differentiation and their interaction with tumor cells. It has been reported that these cells, in some cases, may favor tumor growth (Prantl et al. 2010), or quite the opposite, inhibiting the the proliferation of cancer cells (Cousin et al. 2009). Some studies, carried out on primary tumor cells, conclude that ASCs can induce tumor cell growth in the presence of active tumor cells but not mitoticly inactive ones (Zimmerlin et al. 2011). Eterno et al. (2014) showed that ASCs may support the proliferation of breast cancer cells through the activation of HGF/c-Met/beta-catenin axis, which is very important in the light of increased breast cancer recurrence rates after fat grafts. These contradictory results generate the need for more detailed molecular characterization of adipose derived stem cells. Unfortunately, information relating to undesirable ASCs differentiation is very limited. The only described case regards the appearance of cysts and microcalcifications in 4 of 70 patients undergoing breast reconstruction using lipoaspiration enriched by SVF (Yoshimura et al. 2008). This may be due to the fact that such a phenomenon is rare or has not been fully investigated. Further studies are necessary to elucidate the mechanism of stem cell transformation, which will greatly contribute to cell fate regulation and inhibition of oncogenesis in different organs. Summarizing, it is very important to be aware that stem cell therapies, despite the great hope, also poses weakness and limitation. We have to isolate and culture immune-specific and homogenous stem cell populations, improve the efficiency of differentiation into target cells, establish an optimal stage of transplantation and improve the post-transplantation migration and regeneration rate (Bajek et al. 2014; Lim et al. 2011).

## Conclusion

Although, there are dozens of clinical studies using ASCs, there is an apparent lack of reports that unambiguously confirm the effectiveness of this type of cell therapy. Still, many important questions remain before effective clinical applications of ASCs therapies for humans arise. Nevertheless, the clinical use of ASCs is undeniably very exciting and draws a lot of attention.
